# A “child-friendly” hospital: a difficult definition

**DOI:** 10.1186/1824-7288-41-S1-A39

**Published:** 2015-09-24

**Authors:** Gian Paolo Salvioli

**Affiliations:** 1 Professor Emeritus of Paediatrics, Alma Mater Studiorum – University of Bologna, 40126 Bologna (BO), Italy

## 

It would be quite interesting and instructive to sketch the history of how children's hospitals came to be established. Our first thought goes to the “Ospedale degli Innocenti” in Florence and the establishment of orphanages, which for many years were the only available facilities, but which took in only orphans and children abandoned by their parents. Until paediatrics became an independent branch, also in terms of teaching at the university level with the creation of the first chair in Rome, in the early 1900s, held by Professor Concetti, the beds for hospitalized children were in the Internal Medicine and the General Surgery Wards.

Following the example of the first paediatric clinics established in the late 1800s in France, in Paris, the first children's hospitals were founded also in Italy, in Rome, Palermo, Bologna, Naples, Genoa and gradually in other cities, thanks to bequests from wealthy members of the aristocracy and public and private benefactors.

The technological progress and the development of welfare assistance in the 1960/70s, the scientific commitment of paediatricians and the affirmation of their role in the care of children in the development stage led to the gradual spread of paediatric and neonatology wards in our country.

However, significant differences continue to exist between regions even today and, furthermore, the economic crisis that has been affecting the country for years now is leading to a downgrading of paediatric and neonatology wards, where head physician positions are not filled again upon vacancy and which are merged with adult care wards. 

It would be preferable to cut and downsize the positions of the administrative staff rather than those of the doctors and nursing staff.

Another critical issue remains in terms of the improper hospitalization of children and adolescents in general medicine or specialized (otolaryngology, ophthalmology, dermatology, orthopaedics, etc.) wards for adults.

A “child-friendly” hospital must of course offer surroundings suited to the age of the young patients, from birth to adolescence, such as areas for mothers who are nursing or who have to assist their hospitalized child, a games room, a schoolroom, but above all the facility must allow and ensure the “care” of these young patients, involving their parents and allowing them to remain close to their children.

Moreover, doctors must be freed from the continuous and unrelenting administrative procedures thrust upon them (meetings on budgets, on the various audits, etc.) and given more time to devote to one of the most sensitive aspects of our work: communication with patients and their families.

Ultimately, we are simply re-inventing the wheel; already the ancient Romans reaffirmed the principle that all those involved in childcare should observe: “Maxima debetur puero reverentia!”

